# Supplementary figures and images for: Sensitivity of yeast to lithium chloride connects the activity of YTA6 and YPR096C to translation of structured mRNAs
Source: PLoS One. 2020 Jul 8;15(7):e0235033. doi: 10.1371/journal.pone.0235033 (PMC7343135; doi:10.1371/journal.pone.0235033)

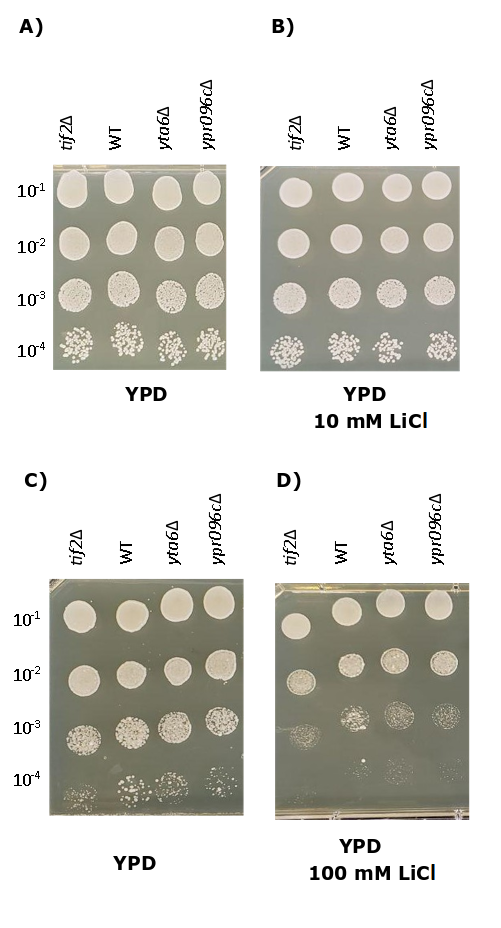

Supplement: S1 Fig — No increased LiCl sensitivity was observed for deletion mutant strains for YTA6 and YPR096C in media containing glucose as a carbon source. Spot test analysis was repeated at least three times (n ≥ 3) with similar outcomes. (TIF) [file pone.0235033.s001.tif]

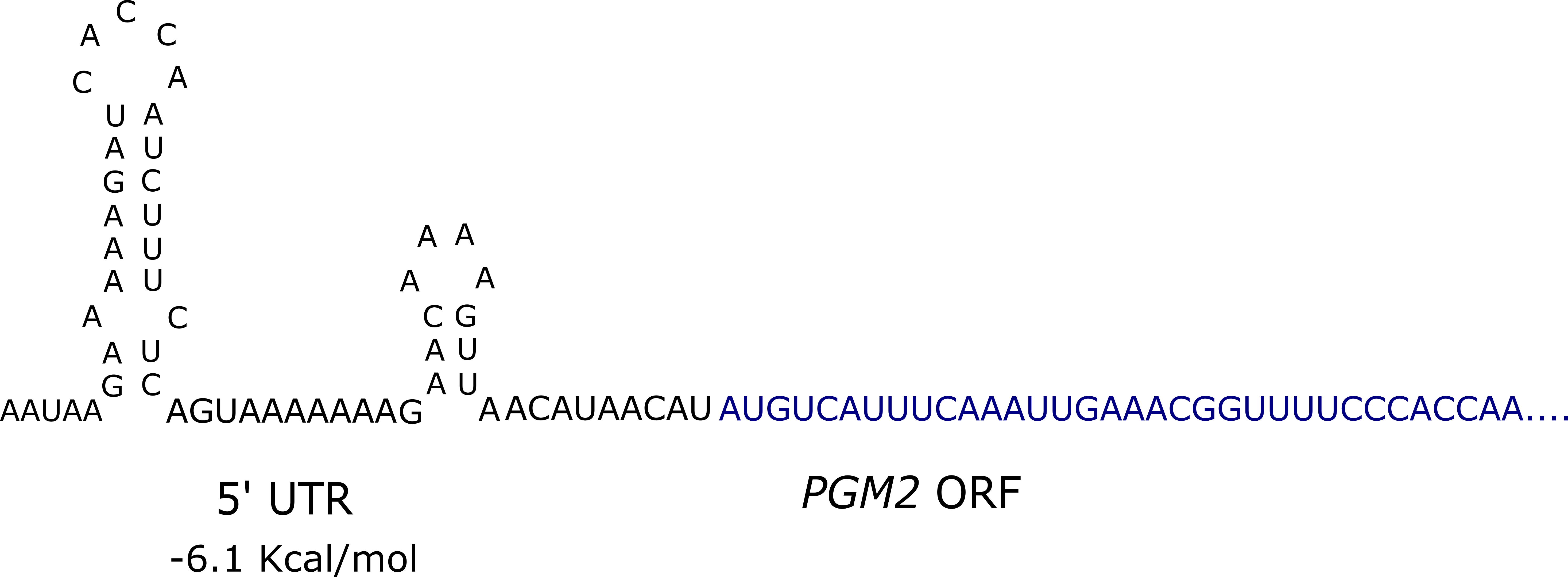

Supplement: S2 Fig — Unlike most yeast ORFs, the 5’ UTR of PGM2 is thought to be structured (38). (TIF) [file pone.0235033.s002.tif]
