# Supplementary material for: Sensitivity of yeast to lithium chloride connects the activity of YTA6 and YPR096C to translation of structured mRNAs
Source: PLoS One. 2020 Jul 8;15(7):e0235033. doi: 10.1371/journal.pone.0235033 (PMC7343135; doi:10.1371/journal.pone.0235033)
Supplement: S1 Table — Each experiment was repeated at least three times (n ≥ 3). (DOCX) [file pone.0235033.s003.docx]

**Table S1. qRT-PCR raw data for different strains with and without LiCl treatment.** Each experiment was repeated at least three times (n ≥ 3).

| **Strain** | **LiCl treatment** | **cq mean (PGM2)** | **cq mean (PGK1)** | **Δcq** | **ΔΔcq** | **Relative expression** | **Standard deviation** |
| --- | --- | --- | --- | --- | --- | --- | --- |
| WT | - | 20.59 | 18.16 | 2.43 | 0 | 1 | 0.046 |
| *YTA6Δ* | - | 21.62 | 19.16 | 2.46 | 0.03 | 0.979420298 | 0.022 |
| *YPR096CΔ* | - | 20.52 | 17.77 | 2.75 | 0.32 | 0.801069878 | 0.018 |
| WT | + | 20.77 | 19.16 | 1.61 | -0.82 | 1.765405993 | 0.015 |
| *YTA6Δ* | + | 20.95 | 19.23 | 1.72 | -0.71 | 1.635804117 | 0.019 |
| *YPR096CΔ* | + | 20.93 | 19.16 | 1.77 | -0.66 | 1.580082624 | 0.034 |
