# Supplementary material for: Sensitivity of yeast to lithium chloride connects the activity of YTA6 and YPR096C to translation of structured mRNAs
Source: PLoS One. 2020 Jul 8;15(7):e0235033. doi: 10.1371/journal.pone.0235033 (PMC7343135; doi:10.1371/journal.pone.0235033)
Supplement: S2 Table — (DOCX) [file pone.0235033.s004.docx]

**Table S2. List of mutant strains in gene expression and random arrays.**

| **Genes found in the gene expression array** | | | **Genes found in the random array** | | |
| --- | --- | --- | --- | --- | --- |
| YLR085C | YER069W | YJL130C | | YMR105C |  |
| YER088C | YDR496C | YAL005C | | YMR105C |  |
| YPL086C | YEL066W | YAL017W | | YMR105C |  |
| YFL031W | YER117W | YAL036C | | YMR119W-A |  |
| YMR172W | YER027C | YBL013W | | YMR120C |  |
| YOL012C | YGL105W | YBL066C | | YMR121C |  |
| YLR451W | YGL236C | YBR025C | | YMR122C |  |
| YGL035C | YGR085C | YBR031W | | YMR123W |  |
| YGL151W | YGR084C | YBR061C | | YMR124W |  |
| YGL013C | YGL115W | YBR101C | | YMR126C |  |
| YJL089W | YGR118W | YBR121C | | YMR127C |  |
| YMR179W | YGR034W | YBR146W | | YMR129W |  |
| YOR047C | YGR027C | YBR181C | | YMR130W |  |
| YGL162W | YGL196W | YBR187W | | YMR132C |  |
| YAL059W | YGL195W | YBR212W | | YMR194W |  |
| YKR059W | YHR189W | YBR294W | | YMR207C |  |
| YJL191W | YHR075C | YBR295W | | YMR209C |  |
| YLR287C-A | YGR201C | YBR296C | | YMR210W |  |
| YNL162W | YHL039W | YBR297W | | YMR214W |  |
| YOL045W | YHL033C | YBR301W | | YMR215W |  |
| YFL034C-A | YIL038C | YCL001W | | YMR216C |  |
| YNL069C | YIL070C | YCL001W-A | | YMR219W |  |
| YML068W | YBR118W | YCL002C | | YMR221C |  |
| YBL032W | YDR477W | YCL006C | | YMR222C |  |
| YGL248W | YIL064W | YCL028W | | YMR223W |  |
| YJL190C | YJL136C | YCL029C | | YMR224C |  |
| YMR012W | YJR014W | YCL037C | | YMR256C |  |
| YNL299W | YIR038C | YCR003W | | YMR258C |  |
| YFR032C-A | YJL162C | YCR026C | | YMR259C |  |
| YDR447C | YJR051W | YCR027C | | YMR261C |  |
| YDL219W | YJR094W-A | YCR028C-A | | YMR262W |  |
| YNL277W | YIR001C | YCR032W | | YMR263W |  |
| YDR378C | YJL073W | YCR033W | | YMR264W |  |
| YLR172C | YLR012C | YCR034W | | YMR265C |  |
| YPL090C | YKL185W | YCR036W | | YMR266W |  |
| YDR450W | YKL202W | YCR037C | | YMR269W |  |
| YMR116C | YKL201C | YCR046C | | YMR271C |  |
| YER081W | YKL198C | YDL083C | | YNL037C |  |
| YCR003W | YJR129C | YDL110C | | YNL040W |  |
| YER056C-A | YKR091W | YDL134C | | YNL041C |  |
| YEL050C | YKR087C | YDL185W | | YNL043C |  |
| YKL074C | YKR084C | YDL188C | | YNL044W |  |
| YLR333C | YKR101W | YDL191W | | YNL045W |  |
| YOR185C | YKL006W | YDL224C | | YNL046W |  |
| YOL080C | YKL156W | YDR025W | | YNL049C |  |
| YDR494W | YKL056C | YDR152W | | YNL050C |  |
| YFR009W | YGR271W | YDR179W-A | | YNL051W |  |
| YNR022C | YNL014W | YDR234W | | YNL052W |  |
| YJR145C | YLR327C | YDR239C | | YNL096C |  |
| YPR043W | YLR264W | YDR241W | | YNL284C |  |
| YLR192C | YLR362W | YDR244W | | YNR041C |  |
| YLR303W | YLR281C | YDR245W | | YNR052C |  |
| YAR018C | YLR388W | YDR247W | | YOR158W |  |
| YHR077C | YLR185W | YDR248C | | YOR197W |  |
| YGL232W | YLR425W | YDR249C | | YOR202W |  |
| YKL081W | YLR441C | YDR250C | | YOR202W |  |
| YLR335W | YMR080C | YDR251W | | YOR202W |  |
| YMR302C | YMR230W | YDR252W | | YOR202W |  |
| YPL048W | YMR226C | YDR275W | | YOR208W |  |
| YPL079W | YMR225C | YDR282C | | YOR209C |  |
| YER131W | YMR020W | YDR293C | | YOR212W |  |
| YBR084C-A | YNL288W | YDR357C | | YOR213C |  |
| YKL204W | YMR269W | YDR358W | | YOR214C |  |
| YGR178C | YNL040W | YDR359C | | YOR215C |  |
| YJR150C | YNL067W | YDR360W | | YOR216C |  |
| WT | YNL063W | YDR363W | | YOR219C |  |
| YLL045C | YNL304W | YDR363W-A | | YOR221C |  |
| YLR344W | YNL223W | YDR368W | | YOR313C |  |
| YOR293W | YNL339C | YDR369C | | YOR314W |  |
| YBR130C | YNL104C | YDR370C | | YOR315W |  |
| YML063W | YML129C | YDR371W | | YOR316C |  |
| YIL052C | YNL096C | YDR372C | | YOR317W |  |
| YCR031C | YNL239W | YDR400W | | YOR318C |  |
| YLR406C | YOR091W | YDR422C | | YOR320C |  |
| YLR039C | YNR020C | YDR432W | | YOR321W |  |
| YDR515W | YOR107W | YDR450W | | YOR322C |  |
| YER074W | YNR045W | YDR500C | | YOR324C |  |
| YLR061W | YOL093W | YEL015W | | YOR327C |  |
| YKL191W | YOR051C | YER049W | | YPL009C |  |
| YML009C | YOL027C | YER153C | | YPL013C |  |
| YMR143W | YOL114C | YFR001W | | YPL127C |  |
| YOR182C | YOL039W | YGL049C | | YPL197C |  |
| YDL037C | YPL081W | YGL070C | | YPL203W |  |
| YIL133C | YOR252W | YGR010W | | YPL226W |  |
| YDL184C | YOR242C | YGR011W | | YPL239W |  |
| YMR129W | YOR354C | YGR012W | | YPL240C |  |
| YDR502C | YPL208W | YGR014W | | YPR014C |  |
| YPL106C | YPL119C | YGR015C | | YPR028W |  |
| YDR116C | YOR276W | YGR016W | | YPR057W |  |
| YKL068W | YPL009C | YGR017W | | YPR075C |  |
| YLR325C | YOR303W | YGR018C | | YPR076W |  |
| YLR398C | YGL049C | YGR019W | | YPR077C |  |
| YOR312C | YFL001W | YGR021W | | YPR078C |  |
| .YPR132W | YPR111W | YGR022C | | YPR079W |  |
| YDL088C | YBR267W | YGR054W | | YPR084W |  |
| YDL175C | YBR261C | YGR054W | | YPR089W |  |
| YBR189W | YDL001W | YGR131W | | YPR090W |  |
| YHR121W | YDL130W-A | YGR132C | | YPR092W |  |
| YMR171C | YBR280C | YGR133W | | YPR093C |  |
| YGR276C | YBR271W | YGR134W | | YPR095C |  |
| YER091C | YCR021C | YGR135W | | YPR100W |  |
| YAL029C | YDL048C | YGR136W | | YPR152C |  |
| YBL087C | YCR059C | YGR137W | | YPR197C |  |
| YDR312W | YCL011C | YGR138C | |  |  |
| YHR021C | YDR198C | YGR139W | |  |  |
| YKL167C | YDR333C | YGR141W | |  |  |
| YML026C | YDR207C | YGR142W | |  |  |
| YOR133W | YDR206W | YGR148C | |  |  |
| YPL198W | YDR101C | YHR010W | |  |  |
| YPR132W | YDR098C | YHR086W | |  |  |
| YFR031C-A | YDL210W | YHR087W | |  |  |
| YDL081C | YDR225W | YHR092C | |  |  |
| YDL160C | YDR117C | YHR093W | |  |  |
| YDL133C-A | YDL229W | YHR094C | |  |  |
| YNL087W | YDR001C | YHR095W | |  |  |
| YJL051W | YDR120C | YHR096C | |  |  |
| YJR137C | YDR257C | YHR097C | |  |  |
| YNL323W | YDR007W | YHR103W | |  |  |
| YBL072C | YDR156W | YHR104W | |  |  |
| YGL031C | YDR282C | YHR105W | |  |  |
| YHR203C | YDR169C | YHR202W | |  |  |
| YLR107W | YDR159W | YHR203C | |  |  |
| YOR017W | YER151C | YHR204W | |  |  |
| YBR048W | YEL011W | YHR206W | |  |  |
| YDL130W | YFL023W | YHR207C | |  |  |
| YDR471W | YFL034W | YHR209W | |  |  |
| YNL001W | YER007C-A | YHR210C | |  |  |
| YKR094C | YER002W | YIL001W | |  |  |
| YOL121C | YDR535C | YIL002C | |  |  |
| YLR434C | YER035W | YIL005W | |  |  |
| YDR363W | YGL208W | YIL006W | |  |  |
| YHR034C | YFR015C | YIL018W | |  |  |
| YGL222C | YGR053C | YIL074C | |  |  |
| YKL130C | YGR081C | YJL092W | |  |  |
| YMR188C | YGL173C | YJL106W | |  |  |
| YFR049W | YGL043W | YJL107C | |  |  |
| YGL147C | YHR047C | YJL117W | |  |  |
| YDL082W | YGR173W | YJL119C | |  |  |
| YDL136W | YHL034C | YJL120W | |  |  |
| YKL205W | YIR009W | YJL138C | |  |  |
| YMR190C | YJL131C | YJL151C | |  |  |
| YBL079W | YJR008W | YJL152W | |  |  |
| YIL079C | YJL122W | YJL153C | |  |  |
| YGR148C | YIL093C | YJL154C | |  |  |
| YLR048W | YJL141C | YJL155C | |  |  |
| YOR234C | YJL137C | YJL177W | |  |  |
| YOL041C | YIL110W | YJR034W | |  |  |
| YPR129W | YIL096C | YJR043C | |  |  |
| YJR047C | YJL148W | YJR047C | |  |  |
| YOL115W | YJL164C | YJR048W | |  |  |
| YLR448W | YJL160C | YJR049C | |  |  |
| YJR148W | YJL158C | YJR050W | |  |  |
| YDR382W | YJL187C | YJR066W | |  |  |
| YGR214W | YIL162W | YKL003C | |  |  |
| YKR026C | YKR058W | YKL127W | |  |  |
| YNL302C | YJR126C | YKR036C | |  |  |
| YPR042C | YKL109W | YKR039W | |  |  |
| YIL071C | YKR024C | YKR040C | |  |  |
| YIL103W | YKL139W | YKR041W | |  |  |
| YPL183W-A | YKL166C | YKR042W | |  |  |
| YKL001C | YLR343W | YKR043C | |  |  |
| YDR385W | YLR221C | YKR044W | |  |  |
| YGR072W | YLR003C | YKR045C | |  |  |
| YKR057W | YLR262C-A | YKR046C | |  |  |
| YLR367W | YLR258W | YKR047W | |  |  |
| YNL301C | YLR137W | YKR048C | |  |  |
| YPL220W | YLR150W | YLL028W | |  |  |
| YOR302W | YLR149C | YLL029W | |  |  |
| YIL148W | YLR289W | YLL032C | |  |  |
| YDL061C | YMR186W | YLL038C | |  |  |
| YER176W | YML007W | YLL039C | |  |  |
| YOL137W | YML109W | YLL040C | |  |  |
| YJL124C | YML028W | YLL041C | |  |  |
| YCL009C | YMR139W | YLL042C | |  |  |
| YBR057C | YNL141W | YLL043W | |  |  |
| YJL176C | YNL255C | YLL044W | |  |  |
| YMR307W | YNL031C | YLL045C | |  |  |
| YEL048C | YMR247C | YLR296W | |  |  |
| YJL095W | YNL300W | YLR297W | |  |  |
| YKR020W | YMR282C | YLR299W | |  |  |
| YNL171C | YMR273C | YLR300W | |  |  |
| YOR115C | YNL197C | YLR303W | |  |  |
| YPL069C | YNL307C | YLR306W | |  |  |
| YOL006C | YNL081C | YLR307W | |  |  |
| YOR028C | YMR297W | YLR309C | |  |  |
| YPR030W | YNL229C | YLR311C | |  |  |
| YBR030W | YNL227C | YLR312C | |  |  |
| YBR026C | YNL224C | YLR313C | |  |  |
| YBL024W | YNR048W | YLR401C | |  |  |
| YBR034C | YOR035C | YLR402W | |  |  |
| YAL040C | YOL001W | YLR404W | |  |  |
| YBR064W | YOL114C | YLR405W | |  |  |
| YBR062C | YOR083W | YLR406C | |  |  |
| YBR186W | YOR078W | YLR407W | |  |  |
| YBR185C | YOL031C | YLR408C | |  |  |
| YBL104C | YPL052W | YLR410W | |  |  |
| YBR222C | YPL157W | YLR412W | |  |  |
| YBR010W | YPL067C | YLR413W | |  |  |
| YBR009C | YPL184C | YLR414C | |  |  |
| YBL003C | YBR232C | YLR441C | |  |  |
| YCR071C | YBR240C | YML112W | |  |  |
