# Supplementary material for: Sensitivity of yeast to lithium chloride connects the activity of YTA6 and YPR096C to translation of structured mRNAs
Source: PLoS One. 2020 Jul 8;15(7):e0235033. doi: 10.1371/journal.pone.0235033 (PMC7343135; doi:10.1371/journal.pone.0235033)
Supplement: S3 Table — (DOCX) [file pone.0235033.s005.docx]

**Table S3. List of negative genetic interactions (nGIs) for YTA6 and YPR096C (no LiCl in media).**

| **SGA hits for *YTA6*** | **SGA hits for *YPR096C*** |
| --- | --- |
| YLR061W | YOR078W |
| YDR159W | YOR091W |
| YBR267W | YDL160C |
| YPR043W | YDR378C |
| YNL197C | YDR159W |
| YPL079W | YMR143W |
| YPL090C | YBR189W |
|  | YBR267W |
