# Supplementary material for: Sensitivity of yeast to lithium chloride connects the activity of YTA6 and YPR096C to translation of structured mRNAs
Source: PLoS One. 2020 Jul 8;15(7):e0235033. doi: 10.1371/journal.pone.0235033 (PMC7343135; doi:10.1371/journal.pone.0235033)

Immunoblots were visualized with chemiluminescent substrates (Bio-Rad®) on a Vilber Lourmat gel doc Fusion FX5-XT (Vilber®). Densitometry analysis was carried out using the FUSION FX software (Vilber®)

The figure panel was generated from this orinigal image of the blot.

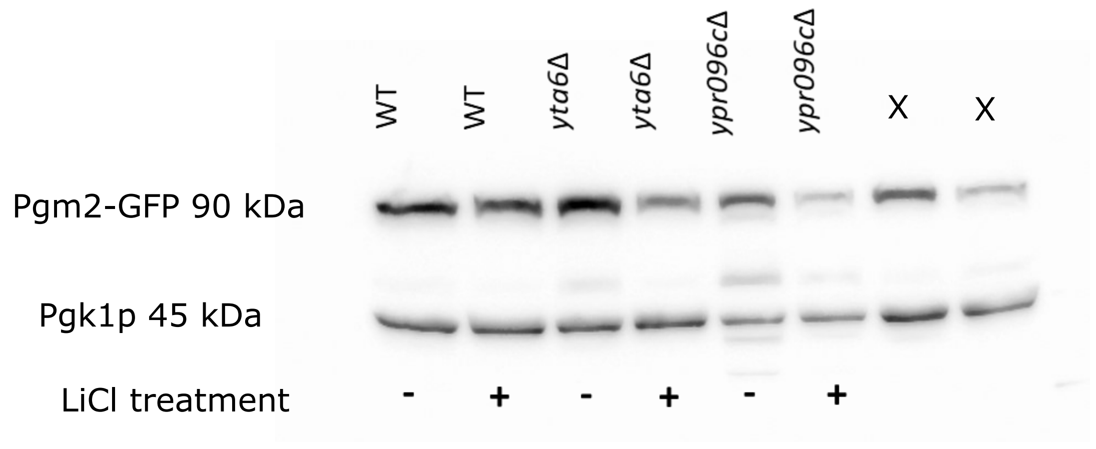

figure on the lefft is incubated with GFP antiboDy and firgure on the right is the same membrane after wash and incubation with Pgk1 antibody

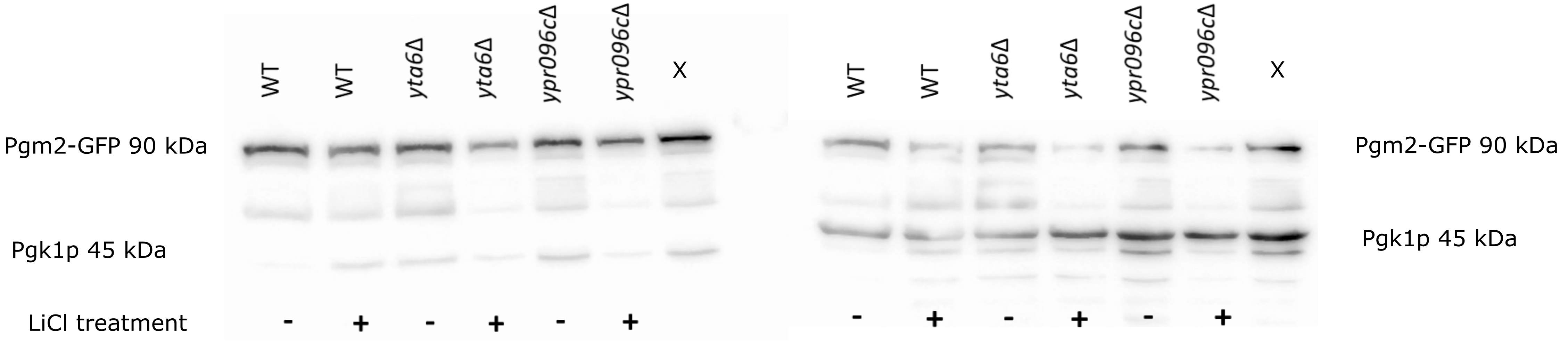

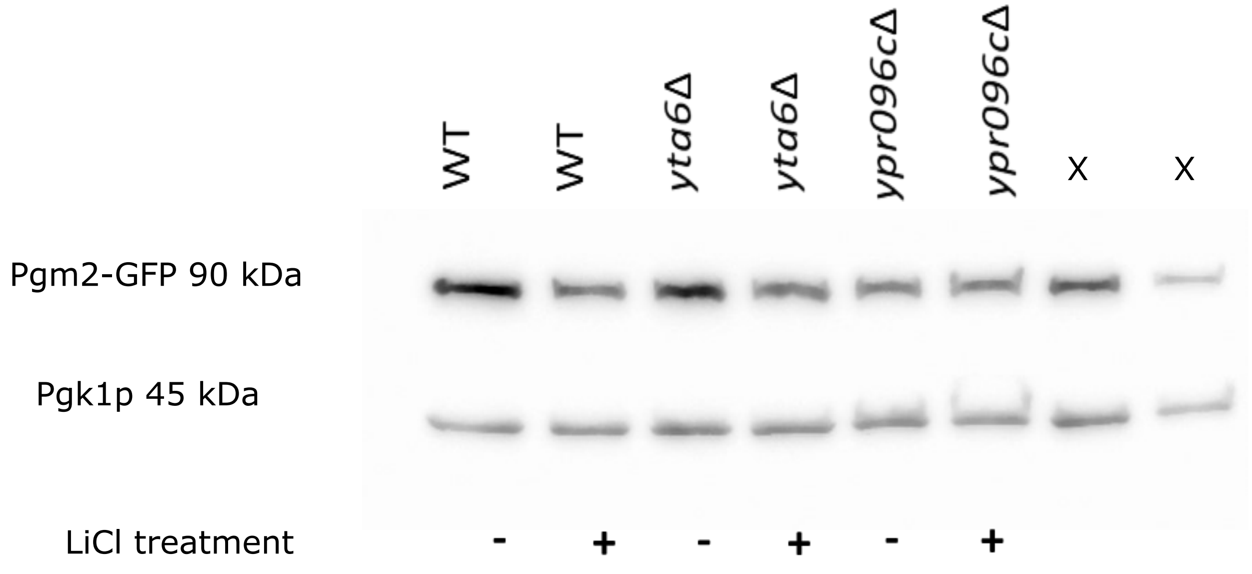

Supplement: S1 Raw Images — (PDF) [file pone.0235033.s006.pdf]
