# Supplementary material for: Sensitivity of yeast to lithium chloride connects the activity of YTA6 and YPR096C to translation of structured mRNAs
Source: PLoS One. 2020 Jul 8;15(7):e0235033. doi: 10.1371/journal.pone.0235033 (PMC7343135; doi:10.1371/journal.pone.0235033)
Supplement: Attachment — Submitted filename: Response to Reviewers.docx [file pone.0235033.s007.docx]

**Responses to Editor and Reviewers comments**

We would like to start by thanking Drs. AJ Lusting, CA Masuda and unanimous reviewer #1 for their invaluable time and comments to improve the quality of this manuscript.

**Editor’s comments:**

- *As Reviewer 2 points out, LiCl also has an interaction in galactose metabolism, independent of toxicity. The consideration of galactose metabolism intermediates that are likely accumulate in yeast cells creating a large amount of phenotypes may be due to specific galactose-1-phosphate accumulation, already known as part of toxicity of  galactosemia. Since there is a second pathway that has not been considered, discussion of that pathway is required. Add appropriate references.*

**Our response:** The text is now modified accordingly.

- *Testing of the alternative pathway per the suggestions of Reviewer 2 is required: 1) control experiments testing lithium toxicity in using media that use other carbon sources (e.g. glucose, glycerol, lactate, etc); 2) test the effect of the gal1 gene deletion on the growth tests of the mutant trains. Test the alternative hypothesis by suppression through deletion of YTA6 and YPR096C.*

**Our response:** Both suggested experiments are now performed and the results are reported. Please refer to our answer to reviewer #2 major comment #1.

- *Cell size as an assay of fitness is flawed since it could be due to a higher cell death rate or a slower growth rate. These paramaters need to be determined independently.*

**Our response**: We agree with this comment. Although cell death and growth rate can both represent sensitivity, they do not signify the same parameter. In the current study, general cell sensitivity serves as a starting point for follow-up analyses. We feel the outcome of this experiment will not affect the conclusions of the current study.

- *Present the results of non-treated cells.*

**Our response:** The text is now modified accordingly (Fig 3).

- *The PGM2 hairpin hypothesis requires more controls including  testing a) the effect of the hairpin on translational assays and b) the absence of the hairpin on the suppression of the phenotype.*

**Our response:** Suggested experiments are now performed. We now have a new b-gal construct with and without *PGM2* hairpin. The results are shown in modified Figure 4 panels A and B.

- *The authors need to clarify the potential of non-isogenicity on the nGI results.*

**Our response:** We agree that such screens have inherent bias. Please refer to our answer to the same point raised by Reviewer #2, under major concerns, point 5. We have used a random plate to estimate the overall rate of GIs for a target gene in our hands. In this case we can drive more meaningful P-values. For clarity, the description of the control plate is modified.

- *Statistical analysis requires a description in methods.*

**Our response:** The text is now modified accordingly.

- *Figure 6 is difficult to see (see the AE comments below).*

**Our response:** The resolution is now improved.

- The major textual problem is the over-comparison with the use of Li in bi-polar disorder, as noted by Reviewer 1.  This may be mentioned only briefly as it is peripheral to the actual study

**Our response:** The text is now modified accordingly.

- *Reviewer 2 has more specialized knowledge essential for the interpretation of the data. Reviewer 1 focused on the presentation.*

*The AE reading of the manuscripts found numerous additional problem. First, Figures 1 and 7 show spot assays that are strips of data. The nature of the splicing is not mentioned in the text and it is unknown whether they are derived from one or more plate. The original data must be presented to the Reviewers for all of the data by the new PLOS One data presentation rules. Second, the other Figures would be assisted by a diagram of the assay shown in that Figure. Third, the statistical tests frequire exact n values and methods in the Figure Legends. Fourth, are the cells that are the origin of the quantitative cell counting independent colonies from independent cells?. If not, this must be repeated with colonies from independent cells that may require a larger sample size.  Fifth, when data are non-statistically significant, they should not be shown in any Figure, including the supplement. Sixth, terms such as gene expression and protein translation are muddled. These need to be fixed. Seventh, the references are incomplete. Please correct carefully. Eighth,  spelling errors detract from the coherent reading of the manuscript.  The authors should have an independent individual unassociated with the paper read the manuscript for spelling and grammar.*

**Our response:** The text is now modified accordingly. We have kept some of the non-statistically significant data, only when a comparison is needed. For example, growth under different conditions.

**Reviewers’ Comments to the Author**

*Reviewer #1: This is a concise piece of work on a physiological target function of LiCl toxicity in yeast. The experiments are properly conducted and the results are reasonably interpreted. The presentation is, however, somewhat misleading. More than half (six lines out of ten) of the Abstract describes the link between LiCl and bipolar disorder, although the work has very little to do with the disease. This disproportional presentation is confusing and misleading for general readership. It may be acceptable to mention bipolar disorder in the Introduction, although I recommend to curtail the part from the Abstract.*

**Our response:** The abstract and the text is modified accordingly.

*Typos or errors:
l.82
Insert a space after his5.
l.130, l.166,
Capitalize c of Licl.
l.180-188
This section should be in the Introduction, and therefore should be removed.
l.191
TIF2 should be Italicized.
l.201
Insert a space between 10 and mM.
l.218 and Fig.1 and 7
Ypgal or YPgal (l.107)? Stick to the same nomenclature.
l.356
EAP1, not EAp1.
l.356
The legend mentions dhh1D but the corresponding strain is tif2D in the Fig.7. Which is correct?*

**Our response:** The text is modified accordingly.

**Reviewer #2:**
Major concerns:
*1- The authors argue that effects observed by LiCl treatment regards only lithium toxicity but, in fact, Masuda et al 2001,2008 demonstrated that there is a potent interaction of LiCl treatment and galactose metabolism. By blocking PGM2 activity with lithium, galactose metabolism intermediates accumulate in yeast cells and a large amount of phenotypes are due to specific galactose-1-phosphate accumulation, a molecule already known as part of toxicity of a genetic disease called classic galactosemia. We cannot exclude the hypothesis presented by the authors that lithium is (directly) inducing a protein translation problem, but at this point they cannot exclude that the translation problem is being induced by galactose-1-phosphate (or other intermediary metabolite of the galactose pathway) accumulation neither. No comment about the galactose-1-phosphate toxicity was presented in this manuscript. Because previous work has observed that most phenotypes under these condition can be suppressed by the galactokinase gene GAL1 deletion, authors should at least comment on the hypothesis, but preferably, test it. Suggestions for this test are: 1) control experiments testing lithium toxicity in other media containing other carbon sources (e.g. glucose, glycerol, lactate, etc); 2) test the effect of the gal1 gene deletion on the growth tests of the mutant trains. If the deletion of YTA6 and YPR096C can be completely suppressed by gal1 deletion, it would favor the hypothesis that the toxicity modulated by the presence of these genes is more related to galactose-1-phosphate accumulation than to lithium toxicity directly*.

**Our response:** We completely agree with this comment. It is a big oversight from our end. We have now performed both suggested experiments. The results are shown in Figure 1D and Figure 4 A and B. In brief, we believe that the influence of LiCl on translation in through previously reported galactose metabolism. The text is modified accordingly. Considering this comment and the new data, the title of the manuscript is now modified.

*2- In order to claim that the effect of the deletions of YTA6 and YPR096C on lithium/galactose toxicity is really due to the impact on PGM2 translation, I would suggest authors to: 1) test the actual PGM2 mRNA hairpin on the translational assays used in this work; 2) test the expression of a PGM2 gene allele without the hairpin – I would expect that this allele would suppress the effect of the deletions.*

**Our response:** We now have a new b-gal reporter construct with and without *PGM2* hairpin. The results are shown in new Figure 4A and B. Reduction of b-gal is linked to the presence of the *PGM2* hairpin on the mRNA.

*3- The quality of the presented figures is bad, some are almost impossible to visualize (especially figure 6), please increase quality for publication.*

**Our response:** The manuscript is modified accordingly.

*4- Usually, yeast spot growth assays are presented as one photograph of each plate containing all the strains that are to be compared. It makes the comparison of relative growth rate more straight-forward and convincing.*

**Our response:** The new Figure 1 is modified accordingly.

*5- The nGI screening present a bias as authors crossed the mutants yta6Δ and ypr0963cΔ to a called “gene expression library”, thus enhancing the chances of enrichment in protein biosynthesis interactions. So, I am not sure whether the enrichment of the class observed is a good indicator in this case. Also, since the screening was performed with a subset of the entire library, authors should list all the mutants included in the screening.*

**Our response:** We agree with this comment. Although this type of enriched screening and its modified versions have been commonly used by us (e.g. Alamgir et al BMC Genomics, 2008; Samanfar et al Mol Biosyst, 2013; Gagarinova et al Cell Rep, 2016; etc) and others (e.g. Laribee et al 2007, PNAS; Collins et al Nature, 2007; Roguev et al Science, 2008; Zheng et al Mol Syst Biol, 2010; etc) they all have certain inherent bias associated with them. In our case we always use a random set of mutants (384 strains) as a control plate to estimate the overall rate of GIs for a target gene in our hands. In this case we can drive more meaningful P-values. For clarity, the description of the control plate is modified.

*6- Some of the arguments used during the manuscript are missing references, and some are wrongly interpreted from the literature. For example, in the lines 35/36 authors argue that LiCl reduces PGM2 expression but Masuda et al 2001 shows that lithium treatment increases the mRNA levels of PGM2. In lines 255-257 the group argue that deletion of TIF2 reduces PGM2 expression in response to LiCl accordingly to Montero-lomeli et al., 2002 but this data does not exist in this publication.*

**Our response:** The manuscript is modified accordingly.

*7- Please recheck the references section of your work. Many references are lacking informations like journal name and/or DOI. In line 173 there is a reference missing from the list (Memarian et al., 2007). In line 191 the reference Bro et al., 2003 is actually Montero-Lomeli et al., 2003.*

**Our response:** The manuscript is modified accordingly.

*8- No statistical analysis details are presented in the work. Although differences in data are clear, please indicate in the method section the type of test used in Figure 4 and perform statistical analysis for the rest of the data.*

**Our response:** The manuscript is modified accordingly.

**Minor concerns:**
*- It would be interesting if authors could present the result of non-treated cells on figure 3b to observe any effect of the deletions on the basal expression of PGM2 gene.*

**Our response:** The suggested experiment is performed and now included in Figure 3.

*- Because of the way the quantitative growth experiment was performed, it is impossible to discern whether the effect of the deletion of YTA6 and YPR096C genes is increasing lethality (cytotoxicity) or decreasing growth rates (cytostatic effect). It would be interesting to discern between these effects performing some viability assays since the figure 2 result shows that overexpression of genes lead to a colony number higher than the WT strain, suggesting a decreased cytotoxicity.*

**Our response:** We agree with this comment. In the current study, general cell sensitivity serves as a starting point for follow-up analyses. We feel the outcome of this experiment will not affect the conclusions of the current study, but plan to include it in our future work. Also we discovered an issue with normalization of our overexpression genes in Figure 2; this is now corrected.

*- The term gene expression is usually used to address gene transcription, not so much for effect in translation. Although I do not consider the actual (in this manuscript) use of the term wrong, I suggest being more precise in describing the phenotypes observed, even in the title, and better establish that the hypothesis is that these genes (and lithium?) affect the process of translation.*

**Our response:** The manuscript is modified accordingly. The title is also modified.

*- For better understanding, improve the description of the method for colony counting in the methods section.*

**Our response:** The manuscript is modified accordingly.

*- The housekeeping gene used to normalize qRT-PCR is always an issue, and I this case PGK1 was used. Is this a good housekeeping gene to this context? Have you tried others such as ACT1 or TAF10?*

**Our response:** This is an interesting point. We have not used additional housekeeping genes but would be interesting to examine a second gene in future.

*- Add references to the arguments in lines 41-42, 42-43, 265 (for TIF2 as control),348-349.*

**Our response:** The manuscript is modified accordingly.

*- Period between lines 47-52 is difficult to understand.*

**Our response:** The manuscript is modified accordingly.

*- Misspelling in lines 126 (Reference of krogan et al 2003); 227 (MasudA et al 2001); 245 – legend- PGM2 protein and mRNA content analysis*

**Our response:** The manuscript is modified accordingly.

*- Cite Tong et al., 2001 also in the methods section*

**Our response:** The manuscript is modified accordingly.
