# Supplementary material for: Sensitivity of yeast to lithium chloride connects the activity of YTA6 and YPR096C to translation of structured mRNAs
Source: PLoS One. 2020 Jul 8;15(7):e0235033. doi: 10.1371/journal.pone.0235033 (PMC7343135; doi:10.1371/journal.pone.0235033)
Supplement: Attachment — Submitted filename: Responses to Editor and Reviewers.docx [file pone.0235033.s008.docx]

**Responses to Editor and Reviewers comments**

We would like to start by thanking Drs. AJ Lusting, CA Masuda and the unanimous reviewer #1 for their invaluable time and comments to improve the quality of this manuscript.

**Editor’s comments:**

- *The manuscript has a large number of grammatical mistakes; in particular, the lack of use of articles (e.g, "the"). This problem is present throughout the paper and compromises the ease of reading the manuscript.  This is necessary to meet the standards for publication in PLOS. Please have an individual familiar with publication standards review your grammar.*

**Our response:** The text is now modified accordingly.

- *Specific errors include the definition of NAT (it is not nourseothricin but, rather nourseothricin N-acetyl transferase).*

**Our response:** NAT is now replaced by clonNAT (nourseothricin sulfate).

- *How was the concentration of LiCl determined? What were the effects in different concentration of LiCl> and a clear relationship between context for which methods are involved in which experiments.*

**Our response:** Proper references are now included for the starting concentrations of LiCl. Additional explanation is also provided. Description is added for each method connecting it to the corresponding experiment.

- *Is the statistical basis of the qPCR the MIQE convention? If not, please redo these evaluations according the this convention.*

**Our response:** Yes, it is. The text is now modified to reflect this.

- *Define SDL.*

**Our response:** “SDL” is now removed from the text.

- *Statistical tests should include the sets of values that are being compared (for example in Western blot analysis). Also place n value and statistical test in Figure Legends.*

**Our response:** The text is now modified accordingly.

- *Please define the unconventional use of "fitness". Are you not referring to colony size?*

**Our response:** A description is now added in the Materials and methods section. In Genetic Interaction analysis, colony size is commonly used as a measure of fitness. For example: Tong et al 2001 Science; Toufighi et al 2011 Nature Methods; Roguev et al 2018 Cold Spring Harb Protoc; etc.

- *Please describe the tests for the functionality of fusion proteins used in this study. Also clarify which experiments used and did not use the fusion protein.*

**Our response:** PCR analysis and LiCl sensitivity was used to confirm the integrity of Pgm2p-GFP strain. The Material and methods section is modified accordingly.

- *The introduction of high copy number genes that complement nulls is not a test of complementation of the gene. Rather, re-integration of a single copy gene is needed to prove that the correct gene has been isolated in plasmids.*

**Our response:** The text is now modified accordingly.

- *Much of the data is presented graphically in Figures 2,3,4,5. An example of each dataset is required in these Figures. Similarly, a subset of the data interactions summarized in Figure 6 that have been uncovered in this paper should be presented. In addition, in a Supplementary Data section, by PLOS One regulations, all of the raw data must be presented so that the data is can be directly assessed.*

**Our response:** An example for each data set is now added. Negative genetic interaction data is now represented in Table S3. An inset for Figure 4A is now included. An inset for Figure 6 is now included.

- *In the spot assays in Figure 1, are all the plates derived from the same microarray plate?*

**Our response:** Yes, each set is spotted on the same plate and grown under the same conditions.

- *The logic behind the interpretation of mutations in the presence of gal1 null alleles must be explained more clearly as noted by Reviewer 2.*

**Our response:** The text is now modified accordingly.

- *The argument for hairpin structure involvement is quite good. Why was the relationship to the growth sensitivity data not tested by conducting hairpin structure experiments in gal null alleles in a subset of experiments?*

**Our response:** *Gal1* deletion analysis was to study the function of *YTA6* and *YPR096C* in the context of LiCl mode of toxicity. It was performed to functionally link sensitivity of *YTA6* and *YPR096C* to the accumulation of toxic intermediate when galactose was used as a carbon source. This is in contrast to other experiments that were mainly concerned with the expression and translatability of mRNAs. For example, *β-galactosidase* activity analyses derived from the constructs that carry different hairpin structures on their 5’-UTRs, including that for *PGM2*, are independent of *GAL1* function. They focus on the ability of mutant strains to translate *β-galactosidase* mRNAs that contain hairpin structures. Similarly, the RNA (qRT-PCR) and protein (western) content analyses were focused on the expression of *PGM2* and not its function. Deletion of *GAL1* is expected to have no consequence in the outcome of these experiments and would not change the conclusion of the study about translation of structured mRNAs.

- *The isolation of interacting genes in some ribosomal protein genes and regulators of translation implicates  the translation process. However,  conclusions the basis of these studies should be in line with the strength of the data.*

**Our response:** The text is now modified to reduce the strength of the concluding sentence regarding GI data.

- *Eliminate the bipolar discussion in the Results.  It is not relevant to this manuscript.*

**Our response:** The text is now modified accordingly.

**Reviewers’ Comments to the Author**

***Reviewer #1:***

1- *As I pointed out in the previous review, the work has very little to do with BD. Furthermore, the results and discussion section should not contain lengthy explanation of the background information. From these reasons, I recommend to eliminate the section from l.174 to l.185.*

**Our response:** The text is now modified accordingly.

**Reviewer #2:**

1- *Abstract: “Reduced activity of phosphoglucomutase in the presence of galactose causes an accumulation of glucose-1-p leading to a number of phenotypes including growth defect.”

Glucose-1-phosphate is not the only metabolite accumulated under this condition. So, it is more accurate to say that “intermediate metabolites of galactose metabolism” accumulates.

And in work of other groups and ours, many of the phenotypes induced by PGM inhibition is mimicked by the deletion of the GAL7 gene. Because of that, we suggest that galactose-1-phosphate (not glucose-1-phosphate) may be the real culprit of these effects. (Reasoning: Galactose-1-phosphate is the only common metabolite downstream of galactokinase (GAL1) that accumulate in both PGM inhibition and GAL7 inhibition conditions)*

**Our response:** The text is now modified accordingly.

2- *Abstract: “In the current study we identify two understudied genes, YTA6 and YPR096C that when deleted increase cell sensitivity to LiCl treatment in yeast.”*

*Specify that the increased sensitivity to lithium is observed only in the presence of galactose.*

**Our response:** The text is now modified accordingly.

3- *Abstract: “…YTA6 and YPR096C exert their activities by influencing PGM2 at the level of translation.”

Suggest to highlight that the mRNA of PGM2 has a structured 5’ UTR to match the title.*

**Our response:** The text is now modified accordingly.

4- *“Lithium chloride (LiCl) has remained an important treatment option for BD for more than ten decades (2,3).”

Correct “ten decades”. The lithium use to treat neurological disorders started with the work of Dr. John Cade in 1949 and only “officially” accepted later on.*

**Our response:** The text is now modified accordingly.

5- *“Phosphoglucomutase is responsible for converting glucose-1-phosphate to glucose-6-phosphate.”

Suggestion: add here the information that lithium is an inhibitor of the enzymatic activity of PGM.*

**Our response:** The text is now modified accordingly.

6- *“When galactose is used as the carbon source, inhibition of phosphoglucomutase by LiCl results in accumulation of glucose-1-phosphate that in turn causes growth defects (11,12).”

Change glucose-1-phosphate to “galactose metabolites intermediates”.*

**Our response:** The text is now modified accordingly.

7- *“…be a rapid loss of ribosomal protein gene (RBG) pre-mRNAs”

RBG is not used afterwards.*

**Our response:** The text is now modified accordingly.

8- *“…LiCl reduces the activity of phosphoglucomutase enzyme leading to the accumulation of galactose-1-phosphate, a toxic intermediate in galactose metabolism.”

Suggestion to change for: … accumulation of intermediate metabolites from the galactose metabolism including galactose-1-phosphate, a toxic intermediate.*

**Our response:** The text is now modified accordingly.

9- *“we generated double gene deletions for YTA6 or YPR096C with GAL1 gene. Deletion of GAL1 gene relieved the sensitivity of gene deletion mutants for YTA6 or YPR096C to LiCl (Fig 1).”

There is no previous reference to the GAL1 gene, what enzyme it encodes and the reasoning behind the GAL1 deletion effect. This experiment needs more contextualization for a non-expert reader to understand these results.*

**Our response:** The text is now modified accordingly. Relative explanation has been added.

10*- “sensitivity of deletions strains for YTA6 or YPR096C to LiCl diminished when glucose was used as a carbon source further connecting the observed LiCl sensitivity for YTA6 and YPR096C deletion strains to galactose metabolism.”

According to the results presented (YPD + 10 mM LiCl), deletions of YTA6 and YPR096C does not cause ANY sensitivity to LiCl in the absence of galactose, not a DIMINISHED sensitivity to galactose.

That said, it would be interesting to check the effect of these deletions to toxic concentrations of LiCl in YPD (100 - 300 mM range). This experiment could either unmask a possible direct effect of lithium independent of galactose metabolism, or further emphasize that the increased sensitivity of these strains to lithium is dependent on its effect on galactose metabolism.*

**Our response:** The text is now modified accordingly. Also, we subjected the yeast gene deletion strains for *YTA6* or *YPR096C* to 100 mM of LiCl in YPD and observed no increased sensitivity for these mutants in comparison to a control strain. This observation is now reported in the text and the results are shown in Fig S1.

11-  *Figures 4 and 5 – Because both figures address the same general question (effect of YTA6 and YPR096C deletion on translation efficiency of mRNAs containing structures), I suggest to either join figures 4 and 5, or transfer the figures 4C and D to figure 5 to isolate on Figure 4 the discussion about PGM2 mRNA.*

**Our response**: New figures are modified accordingly. The new figure 5 now includes the old figure 4C and 4D panels.

12- *Since the nGI and PSA screenings were performed with a subset of the entire Yeast KO library, authors should list all the mutants included in the screenings and identify the groups (gene expression / random groups).*

**Our response**: The list has been added to the supporting information.
