# Supplementary material for: Sensitivity of yeast to lithium chloride connects the activity of YTA6 and YPR096C to translation of structured mRNAs
Source: PLoS One. 2020 Jul 8;15(7):e0235033. doi: 10.1371/journal.pone.0235033 (PMC7343135; doi:10.1371/journal.pone.0235033)
Supplement: Attachment — Submitted filename: Responses to Editor and Reviewers comments.docx [file pone.0235033.s009.docx]

**Responses to Editor and Reviewers comments**

We would like to start by once again thanking Drs. AJ Lusting, CA Masuda and the unanimous reviewer #1 for their invaluable time and comments to improve the quality of this manuscript.

**Editor’s comments:**

*1- First, please provide the alternative interpretation of the data expressed by Reviewer 2 or a rebuttal of that viewpoint.*

**Our response:** The suggested alternative explanation is now included in the text. Lines 372-377.

*2- Second, the AE question regarding the failure to integrate a single copy of the gene to show complementation was not fully addressed. What is the evidence that the expected gene has been cloned?*

**Our response:** Discussion of complementation is now removed from the text and replaced by reporting the phenotypic observations after the introduction of the over-expression plasmid into the corresponding gene deletion mutants. These plasmids were purchased from Thermofisher and their integrity was confirmed by PCR analysis. Lines 81, 82, 195-197, 202 and 203.

*3- Third, while the images are quite good, the pdf is present at limiting resolution. Be certain that you increase the resolution of the figures and possibly the size of the lane designations so that they will be easily visible in the final version.*

**Our response:** Resolution of all figures is now increased.

*4- Fourth, while the authors have addressed most of the issues, the answers to the AE's critique do not specify where in the text (line numbers) specific changes have been made. In the absence of this information, which by convention should be provided by the authors, the AE is forced to go through multiple re-reads to verify that each point has indeed been addressed. The AE requests that this detail be provided to ensure that every question has been answered.*

***Corresponding Associate Editor’s comments from the previous round:***

- *Specific errors include the definition of NAT (it is not nourseothricin but, rather nourseothricin N-acetyl transferase).*

**Our response:** NAT is now replaced by clonNAT (nourseothricin sulfate). **Line 89.**

- *How was the concentration of LiCl determined? What were the effects in different concentration of LiCl> and a clear relationship between context for which methods are involved in which experiments.*

**Our response:** Proper references are now included for the starting concentrations of LiCl. Additional explanation is also provided. Description is added for each method connecting it to the corresponding experiment. **Lines 114-116, 170-171 and 227-228.**

- *Is the statistical basis of the qPCR the MIQE convention? If not, please redo these evaluations according the this convention.*

**Our response:** Yes, it is. The text is now modified to reflect this. **Line 137.**

- *Define SDL.*

**Our response:** The text is now modified accordingly. SDL is removed from line 165 as this form of genetic interaction is not included in the manuscript.

- *Statistical tests should include the sets of values that are being compared (for example in Western blot analysis). Also place n value and statistical test in Figure Legends.*

**Our response:** The text is now modified accordingly. Lines 211, 215, 217, 219, 256-258, 276-280, 301-305, 354-355, and 387-388.

- *Please define the unconventional use of "fitness". Are you not referring to colony size?*

**Our response:** A description is now added in the Materials and methods section. In Genetic Interaction analysis, colony size is commonly used as a measure of fitness. For example: Tong et al 2001 Science; Toufighi et al 2011 Nature Methods; Roguev et al 2018 Cold Spring Harb Protoc; etc. **Lines 162-163.**

- *Please describe the tests for the functionality of fusion proteins used in this study. Also clarify which experiments used and did not use the fusion protein.*

**Our response:** PCR analysis and LiCl sensitivity was used to confirm the integrity of Pgm2p-GFP strain. The Material and methods section is modified accordingly. **Lines 82-85.**

- *Much of the data is presented graphically in Figures 2,3,4,5. An example of each dataset is required in these Figures. Similarly, a subset of the data interactions summarized in Figure 6 that have been uncovered in this paper should be presented. In addition, in a Supplementary Data section, by PLOS One regulations, all of the raw data must be presented so that the data is can be directly assessed.*

**Our response:** Supporting information for each data set is added. An example of each dataset is added to each figure in lines 215, 277, 302 and 355. Table S1, S2 and S3 in lines 544-553 are added as supporting information data and are mentioned in the manuscript in lines 322, 325, 326, and 330. Figure 6, a representative interaction is now included; also see line 355.

- *The logic behind the interpretation of mutations in the presence of gal1 null alleles must be explained more clearly as noted by Reviewer 2.*

**Our response:** The text is now modified accordingly. **Lines 221-222.**

- *The isolation of interacting genes in some ribosomal protein genes and regulators of translation implicates  the translation process. However,  conclusions the basis of these studies should be in line with the strength of the data.*

**Our response:** The text is now modified to reduce the strength of the concluding sentence regarding GI data. Lines **347-349.**

**Reviewer #2:**

*1- line 219: "In yeast, galactose-1-phosphate is encoded by the GAL1 gene."Correct to galactokinase is encoded by the GAL1 gene.*

**Our response:** The text is now modified accordingly. **Line 222.**

*2- lines 363-365: "The fact that YTA6 and YPR096C compensated the same two gene deletions, further connects their activities together in the context of LiCl sensitivity."*

*Although this conclusion is possible (and should be kept in the manuscript), another possibility is that the overexpression of YTA6 and YPR096C would improve PGM2 translation, leading to an increase in PGM2 activity in cells that was shown to confer resistance to lithium in galactose medium (Masuda et al., 2001). According to this hypothesis, if the main cause of toxicity*

*under these conditions is the decrease in PGM activity, the overexpression of YTA6 and YPR096C would be "solving" the original problem and thus making any yeast strain more tolerant to lithium, not only those with related function in the cell.*

*Since at this point we cannot discern between the two hypothesis, I think authors should discuss both possibilities.*

**Our response:** The text is now modified accordingly. **Added to manuscript from lines 369-375**.

*3- line 418: reference # 11.The citation format for this reference is wrong. Please correct this one, and check carefully for other mistakes in the reference.*

**Our response:** The text is now modified accordingly.

*4- Figures quality: Still the quality of the figures presented in this PDF is low, especially Figure 5 that even if we amplify in a PDF reader, some legends are not readable. Possible that this is a problem of conversion to PDF, but be careful when submittingthe final versions of the figure to assure readability in the final version of the manuscript.*

**Our response:** Resolution of all figures is now increased.
